# Radiation and Glands

**Published:** 1905-11-25

**Authors:** 


(*To be continued.*)

### RADIATION AND GLANDS.

It has been shown that $x$-rays have a power in reducing the size of the spleen in leukæmia, and numerous observations have been made which serve to demonstrate that these newer forms of radiation have an active power over other glandular structures. This is a factor which is forcing itself upon observers, sometimes in by no means a pleasant way. It has been observed before in these columns how that the action of the $x$-rays upon the skin has a depressing, even a destructive, effect upon the sebaceous glands of the skin, a useful property of service in the treatment of inveterate acne vulgaris.

Heinecke[1] has showed that brief exposure of rabbits, dogs, and mice to the rays produces a destructive process in the lymph-follicles. With an exposure too slight to affect the skin he was able to cause a distinct destruction of nuclei of the leucocytes in the follicles of the spleen, leading to their almost complete disappearance, while some destruction was noticed in the lymphoid elements of the bone marrow. That this is a direct action of the rays, and not one caused indirectly by the nervous system or blood changes, is shown by Bergonie,[2] who found that glands exposed rapidly became smaller, tenderness and infiltration disappeared, while other glands which did not come under the influence of the rays were not affected.

Various results have been obtained in the treatment of tuberculous glands. Pusey[3] states that though the results have been variable, yet in tuberculous joints, glands, and deep sinuses, there have been marked successes. Bullit[4] has collected 226 cases, and tabulates them thus: 79, or 35 per cent. cured; 92, or 40 per cent. improved; 55, or 25 per cent. unimproved.

Constable Hayes[5] gives two interesting cases. One, a girl, æt. four, was left with glandular abscesses in the neck after scarlet fever. From the jaw to the clavicles the skin was inflamed, and there were numerous discharging sinuses with undermined edges and large masses of granulations behind the angle of the jaw on the left side. Large masses of glands presented under each sternomastoid, and in the submaxillary and submental regions. After six weeks of short exposure daily to the *x*-rays the skin was healthier and the glands greatly reduced in size. Treatment was thus continued from February 27th to May 6th, when it was discontinued. By June 1st the neck appeared normal, except for slight scarring behind the angle of the jaw, no glands being palpable.

In another case, though not so severe, discharging sinuses and glands disappeared after four months' intermittent treatment. Jean Ferrand and Kronchkoll[6] have found similarly that improvement has followed the ray treatment of tubercular cervical adenitis, even though suppurative, and they consider that all such cases should be tried. With such records in respect of lymphoid structures it is not surprising that attempts have been made to reduce the size of enlarged thyroid glands. Two cases of this condition were treated by Stegman.[7] One, a woman æt. 52, was being treated for carcinomatous supraclavicular glands, and it was noticed that a parenchymatous goître of considerable size lost one quarter of its size after six applications, while at the same time the carcinomatous glands ceased to be palpable. The second, a girl, æt. 21, with a large soft parenchymatous goître, after two exposures lost 3.8 centimetres in circumference of the neck, with relief of the previous dyspnœa.

Pusey has also observed some reduction in size in parenchymatous goîtres, but states that in most cases no benefit accrued. Abbé[8] records a single case of enlarged thyroid with considerable thyroidism successfully treated by the insertion of a tube of radium into the hypertrophied middle lobe. The case was that of a girl, æt. 21, who had a general enlargement of the gland, dyspnœa, palpitation, a pulse-rate of 140, a continual sensation of suffocation on assuming the recumbent position, inability to perform any action requiring exertion, and protrusion of the eyeballs. The treatment consisted in making a median incision into the isthmus under cocaine anæsthesia, and burying a tube containing 10 centigrammes of radium of 300,000 activity, for 24 hours. For a month little happened, but in eight weeks the goître had diminished so as to be scarcely observed. The patient felt quite well, could run upstairs without palpitation, play tennis, and walk several miles. Headache had gone with the exophthalmos, there were no tremors, though some tachycardia remained; pulse 105-120. No other treatment had been used, except attention to the evacuations. Abbé attributes the good result to the action of the radium in either causing a retrograde change in the hyperplasia of the gland, or the possible action upon the ganglia of the sympathetic, and brings evidence to show that the rays of radium may penetrate great distances without refraction, so that even the thoracic and cardiac glands, in his opinion, may be affected.

[1] Münchener, Med. Woch., May 3, 1904. [2] Archiv. d'Elect. Med., April 25, 1905, Jour. Electro. and Radio. May, 1905. [3] Brit. Med. Jour., July 8, 1905. [4] Edin. Med. Jour., April, 1905. [5] Archives of Rönt. Ray, September, 1905. [6] Med. Electro. and Radio., August 1905. [7] Med. Elect. and Radio., August, 1905. [8] Archiv. of Rönt. Ray, March, 1905.

*(To be continued.)*

